# Advancing the Frontiers of Alloys and Composites Through Microstructural Design and Property Tailoring

**DOI:** 10.3390/ma19102015

**Published:** 2026-05-12

**Authors:** Wei Guo

**Affiliations:** State Key Lab of Materials Processing and Die & Mould Technology, School of Materials Science and Engineering, Huazhong University of Science and Technology, Wuhan 430074, China; weiguo@hust.edu.cn

The perpetual drive for enhanced performance, energy efficiency, and sustainability in sectors like aerospace, automotive, and electronics hinges on the development of advanced materials. Alloys and composites, with their remarkable capacity for property tailoring through compositional and microstructural design, stand at the forefront of this endeavor. Recent years have witnessed paradigm shifts, moving from traditional trial-and-error methods toward theory-guided design, and from single-property optimization toward achieving an exceptional balance of multiple, often mutually exclusive, performance metrics. In metallic alloys, the advent of high-entropy alloys (HEAs) has dismantled the conventional single-principal-element design philosophy, opening up a vast compositional landscape with unique properties such as high strength, excellent corrosion resistance, and thermal stability [1,2]. Recent reviews have further highlighted that HEAs are now transcending purely structural applications, with growing interest in their multifunctional properties encompassing magnetic, catalytic, and hydrogen-related functionalities [3]. Simultaneously, in the realm of composites, the integration of advanced manufacturing with computational mechanics has enabled the precise study of process–structure–property linkages, allowing for the prediction and mitigation of failure mechanisms with unprecedented accuracy [4].

Despite this progress, significant knowledge gaps persist, hindering the full realization of these materials’ potential. A critical challenge lies in deciphering the complex, multi-scale relationships between processing parameters, resulting phase constituents, and the final macroscopic properties, particularly under extreme service conditions. For instance, the rapid solidification inherent to additive manufacturing processes like Selective Laser Melting (SLM) creates intricate microstructures and performance outcomes that are difficult to predict using empirical knowledge alone, forming a barrier to the reliable production of high-performance components [5]. Similarly, a long-standing gap exists in understanding the directional dependence of properties, or anisotropy, which is often imparted by manufacturing processes and can significantly compromise structural integrity if not properly accounted for in design [6]. Other gaps include the insufficient exploration of functional properties, such as hydrogen affinity or photocatalytic performance, in new alloy systems like HEAs and metallic glasses. In the context of hydrogen, for example, hydrogen embrittlement remains one of the most complex and unresolved material degradation phenomena, involving intricate interactions between hydrogen and microstructural interfaces across multiple spatial and temporal scales [7]. Furthermore, the difficulty in accurately simulating the failure behavior of composites with manufacturing-induced defects using conventional continuum mechanics persists as a major computational obstacle [8].

This Special Issue, “Alloys and Composites: Structural and Functional Applications, Second Edition” has directly confronted these challenges, assembling 13 cutting-edge studies that illuminate the path from fundamental understanding to practical application. A core theme has been the transition from empirical to predictive methodologies for process optimization. Zhang et al. [5] addressed the challenge of SLM process unreliability by developing a hybrid AHP-WPSO model, achieving over 96% accuracy in recommending process parameters for a high-temperature alloy, thereby providing a robust solution to reduce costly trial-and-error cycles. Such predictive approaches resonate with the broader ICME vision, where data-driven frameworks are increasingly employed to intelligently discover advanced structural metals by integrating multi-scale computational tools with materials informatics [9]. In the domain of composites, the issue of anisotropic material behavior is critically examined from multiple perspectives. Beltran-Zuniga et al. [6] elucidated the fatigue life anisotropy in pipeline steel, quantifying how microstructural features like pearlite banding directly control directional fatigue endurance. Complementing this, Chen et al. [10] proposed a process–structure co-optimization strategy for GFRP automotive components, demonstrating that mapping injection molding histories (fiber orientation, residual stress) onto structural simulations is essential for reliable performance evaluation, effectively bridging the accuracy gap between simulation and real-world testing.

The quest for superior structural properties through novel alloy design and processing is another significant contribution. Li et al. [11] investigated the friction and wear properties of AlCrTiVNb high-entropy alloys, revealing that post-annealing precipitation strengthening and the self-lubricating effect of an FCC phase can drastically improve wear resistance. This work exemplifies the broader trend in HEAs, where the exploration of the immense compositional space continues to yield alloys with exceptional strength–ductility combinations, as systematically reviewed in recent comprehensive assessments of their deformation mechanisms [12]. For medical applications, Xu et al. (Contribution 1) harnessed the twinning-induced plasticity (TWIP) effect in a Ti-15Mo alloy through cyclic deformation and heat treatment, achieving a refined microstructure that delivered an optimized balance of high hardness and excellent wear resistance. The fundamental understanding of such TWIP mechanisms in metastable β-Ti alloys—particularly the interplay between {332}<113> twinning, stress-induced phase transformations, and strain hardening—has been a subject of intense investigation and is critical for designing the next generation of biomedical and structural titanium alloys [13]. This Special Issue has also expanded the functional frontiers of advanced materials. Saksl and Nigutova et al. (Contribution 2) provide an experimental validation of a semi-empirical model to design HEAs with negligible hydrogen absorption, a critical property for hydrogen-resistant structural materials. Looking forward, an in-depth understanding of hydrogen-induced degradation and the design of microstructural interfaces resistant to hydrogen embrittlement will be essential to enable a safe hydrogen economy [14]. Meanwhile, Wei et al. (Contribution 3) functionalized a Zr-based bulk metallic glass with a corrosion-resistant, photocatalytic superhydrophobic coating, demonstrating a path to materials capable of pollutant degradation in harsh environments. Further studies on novel Al-Si-Cu-Ni heat-resistant alloys (Contribution 4) and low-cost, high-ductility dual-phase steels (Contribution 5) reinforce the innovation in processing techniques like squeeze casting and specialized heat treatments to achieve previously unattainable property combinations.

Having highlighted how these studies address existing challenges, the primary focus of this editorial is to chart the most promising avenues for future research. The convergence of physics, data science, and manufacturing will define the next generation of alloys and composites.

## 1. Full Lifecycle Digital Twins Through Integrated Computational Material Engineering (ICME)

The work by Chen et al. [10] and Zhang et al. [5] is a stepping stone toward a fully predictive ICME framework. Future research must strive to create a “digital twin” of the material that spans its entire lifecycle—from alloy design and processing to in-service performance and degradation. This involves the seamless and multi-scale integration of diverse physics-based models: thermodynamic and kinetic calculations for phase prediction, crystal plasticity finite element methods (CPFEM) to capture anisotropic deformation, and peridynamics or phase-field models to simulate damage initiation and evolution, as demonstrated by Wang et al. [4]. A key challenge is integrating these models across length scales, from atomistic dislocations to macroscopic components, while simultaneously feeding in process history data (e.g., thermal profiles, solidification rates) to predict the statistical distribution of properties, not just deterministic values. Machine learning surrogates will be essential to accelerate these computationally heavy multi-physics models for high-throughput optimization. Recent advances in ICME have demonstrated that coupling crystal plasticity formulations with data-driven approaches can effectively capture the complex material behavior under multi-axial loading conditions, providing a proven pathway toward more realistic digital twins [15].

## 2. Generative AI for Inverse Alloy and Composite Design

The paradigm must shift from forward prediction (“what properties will this composition yield?”) to inverse design (“what composition and process will achieve this set of target properties?”). The success of the semi-empirical hydrogen affinity model (Contribution 2) provides a foundation for data-driven approaches. Future work should harness generative deep learning models—such as generative adversarial networks (GANs) or diffusion models—trained on extensive, high-quality experimental and simulated datasets. These models can explore the astronomical compositional space of HEAs or the complex architectural space of composites to identify optimal solutions that simultaneously satisfy multiple property constraints (e.g., high strength, high ductility, and corrosion resistance) while meeting cost and sustainability criteria. The development of such approaches is timely, as the HEA community increasingly recognizes the need to move beyond mechanical optimization toward multifunctional property profiles that combine structural performance with electronic, magnetic, and catalytic functionalities [3]. The key bottleneck is the curation of massive, FAIR-compliant materials databases with sufficient metadata on processing history.

## 3. Intelligent Additive Manufacturing for Hierarchical and Functional Gradient Materials (FGM)

Additive manufacturing offers unparalleled control over spatial material distribution. The next frontier is the “intelligent” fabrication of components with site-specific, hierarchical microstructures. Building on the SLM parameter optimization [5] and fiber orientation control [10], future research should focus on closed-loop systems where real-time in situ monitoring data (melt pool signatures, thermal images) is fed into reinforcement learning agents. These agents would autonomously adjust laser power, scan strategy, or even dynamically mix multiple powder feedstocks to create components with engineered gradients in composition, grain structure, and crystallographic texture. The goal is to produce FGMs where a part’s surface is hard and wear-resistant while its core is tough and compliant, mirroring nature’s designs. This demands groundbreaking work on meta-stable alloy design, such as the Ti-15Mo alloy (Contribution 1), to create new deformation pathways that can be locally activated. Machine learning-driven approaches have recently shown that material-agnostic strategies can successfully predict optimal process conditions across different powder materials, accelerating the development cycle for new alloy systems in additive manufacturing [16].

## 4. Understanding and Engineering Extreme Environment Behavior

Performance under coupled extreme conditions remains a serious challenge. Future studies must move beyond uniaxial, room-temperature, and single-environment testing. This requires novel in situ and operando characterization platforms capable of simultaneously applying mechanical load, high temperature, corrosive media, and irradiation while performing high-resolution imaging and spectroscopy. A fundamental understanding of dynamic microstructural evolution at temporal and spatial extremes is needed. For instance, designing the next generation of heat-resistant aluminum alloys (Contribution 4) or DP steels (Contribution 5) will require elucidating the synergistic effects of creep, oxidation, and thermal fatigue on nano-precipitate coarsening kinetics. For hydrogen technologies, the focus must expand from designing hydrogen-absorbing (Contribution 2) or hydrogen-resistant alloys to understanding the atomistic mechanisms of hydrogen embrittlement across different crystal structures and interfaces under realistic gas pressures and temperatures. Comprehensive reviews have systematically mapped the interaction between hydrogen and critical microstructural interfaces—grain boundaries, twin boundaries, and nano-precipitates—highlighting that the control of these interfaces through advanced thermo-mechanical processing is among the most promising strategies for designing hydrogen-tolerant alloys [17].

In conclusion, this Special Issue showcases a vibrant moment in materials science, where the boundaries of alloy and composite design are being rapidly expanded. The path forward is unequivocally interdisciplinary, demanding a tight symbiosis between domain-driven physical models, data-driven artificial intelligence, and advanced manufacturing. By embracing these future directions, the community can move beyond merely tailoring materials for specific applications toward a new era of on-demand, performance-driven material creation.
